# Gut microbiome and gastric cancer: microbial interactions and therapeutic potential

**DOI:** 10.1186/s13099-025-00729-w

**Published:** 2025-07-26

**Authors:** Maged Tharwat Elghannam, Moataz Hassan Hassanien, Yosry Abdelrahman Ameen, Emad Abdelwahab Turky, Gamal Mohammed ELattar, Ahmed Aly ELRay, Mohammed Darwish ELTalkawy

**Affiliations:** Hepatogastroenterology Department, Theodor Bilharz Research Institute (TBRI), Giza, Egypt

**Keywords:** Gut microbiota, Gastric cancer, Diagnostic implications, Therapeutic potentials

## Abstract

The development of gastric cancer is significantly influenced by the intestinal microbiota, with H. pylori serving as a major risk factor. Through genotoxic effects, persistent inflammation, and metabolic changes, other microbes also play a role. It has been demonstrated that cancer patients and healthy people have different microbiome compositions. Cancer can be inhibited or promoted by the gut microbiota and its metabolites. The relationship between intestinal flora, bacterial extracellular vesicles, and the tumor microenvironment directly affects tumor progression and efficacy of anti-tumor medications, indicating the importance of the tumor microenvironment in tumor survival. Gastrointestinal malignancies may be brought on by the gut microbiome’s dysregulation of non-coding RNA expression. Non-coding RNAs are intriguing avenues for future therapeutic and diagnostic research. The tumor microenvironment is altered by bacterial extracellular vesicles, which may have an effect on immunosuppression, treatment resistance, metastasis, and cancer progression. Further research is required to completely understand the involvement of non-coding RNAs in GI cancers. By modifying drug metabolism and absorption, which have a substantial impact on healing efficacy and serious impact profiles, the dynamic changes in gut microbiota also have a considerable impact on the results of anti-cancer treatment. Improved treatment approaches may arise from a better understanding of the role of the microbiome in gastric malignancies.

## Introduction

Gastric cancer is the fifth most common cause of cancer-related death worldwide and ranks fifth in terms of incidence [1]. At diagnosis, 61% of patients with gastric cancer have advanced disease [2]. There are nearly 1.1 million new cases of GC each year, with approximately 1.1 million new cases of GC are reported each year, and the disease is responsible for about 800,000 deaths, or roughly 7.7% of all cancer-related deaths [3]. Many patients with advanced gastric/gastroesophageal junction cancers still have a poor prognosis despite advancements in treatment [4]. From 2014 to 2020, the United States’ five-year relative survival rates by stage at diagnosis were 75% for localized tumors, 36% for tumors with regional spread, and 7% for patients with distant spread [2]. The prevalence of GC is rising among those under 50 in both low-risk and high-risk areas. This may be due to changes in the gastric microbiome caused by modern lifestyles and the rise in obesity [5].

Approximately one-third of cancers that cause death are digestive system cancers. Infectious agents precipitate at least 15–20% of cancers; tobacco products are linked to 20–30% of cancers, and diet, inactivity, and obesity account for 30 -35% of cancers [6].

There are two different types of GC: diffuse and intestinal [7]. The Correa cascade, comprised of intestinal epithelial metaplasia (IM), erosive gastritis, atrophic gastritis (AG), and normal gastric mucosa, best describes intestinal-type GC. After heterogeneous proliferation, it progresses to invasive carcinoma and GC in situ [8]. While little is known about this process, it is well known that inflammation and Helicobacter pylori (Hp) may contribute to the development of diffuse GC [9].

The entire community of microorganisms that live in the gastrointestinal tract is known as the microbiota, and bacteria make up the majority of this community [10]. Loss of beneficial probionts, reduction in microbiome diversity, and increase in commensal-derived pathobionts are the hallmarks of gut microbiota dysbiosis [11]. Proteobacteria, Firmicutes, Actinobacteria, and Bacteroidetes accounted for 93.5% of the species isolated from humans that were categorized into 12 different phyla [12]. Microbiota helps maintain the integrity of the mucosal barrier, protect against infections, and supply nutrients like vitamins. Furthermore, appropriate immune function depends on the commensal microbiota’s interaction with the mucosal immune system [13]. Vitamin B12, folic acid, vitamin K, riboflavin, biotin, nicotinic acid, pantothenic acid, pyridoxine, and thiamine are among the essential vitamins that it can de novo synthesize [14]. It can also ferment complex carbohydrates, producing metabolites like short chain fatty acids (SCFAs) [15]. By generating a range of bioactive substances, including SCFAs, vitamins, and secondary metabolites, the gut microbiota has recently been recognized as a crucial regulator that profoundly affects stem cell function [16]. We still don’t fully understand the specific makeup of the gastrointestinal microbiota in GC and how these microbial communities change as GC progresses [17–19].

The microbial diversity and abundance in tumor tissues of GC patients are higher, indicating possible connections between stomach microorganisms and cancer [20–22]. Streptococcus, Lactobacillus, Veillonella, Helicobacter, and Prevotella, have been frequently reported in multiple studies and meta-analyses, demonstrating their diagnostic value for GC [23].

Although findings from various studies exhibit considerable variability, 16 S ribosomal RNA (rRNA) gene sequencing which examines both conserved and variable regions of the 16 S rRNA gene, has become essential for taxonomic classification of bacterial genomes and the basis of bacterial diversity research [24, 25].

The 16 S rRNA data, with high throughput, culture independence, and high sensitivity and specificity, offer significant advantages in identifying microbiota associated with GC. However, it has several limitations including time-consumingprocess that involves multiple complex steps, along with the need for specialized equipment and computational resources, thereby limiting its scalability for clinical applications. Also, it cannot differentiate between microorganisms that are.

alive and metabolically active. RNA sequencing offers a more dynamic perspective on microbial activity, offering more advantageous means for exploring the relationship between microorganisms and GC [26].

The Wnt/β-catenin pathway is a pivotal regulator of several biological processes, including cellular proliferation, migration, and tissue homeostasis [27]. It has been proposed that gut microbiota may influence the activity of YAP/TAZ via Wnt/β-catenin signaling, offering fresh perspectives on the role of microbiota in cancer initiation and development [28, 29].

The review will cover the microbial factors, the mechanisms of action, the clinical implications, and finally the interventions and future directions.

### Helicobacter pylori and GC

One of the main risk factors for intestinal GC is known to be Hp infection [30]. GC may develop as a result of microbial dysbiosis [31, 32]. Long and his associates discovered possible links between cancer and the gut microbiota. Using Mendelian Randomization study, they found a positive causal direction with cases of gastric cancer [33]. Chronic inflammation of the stomach mucosa, oxyntic cell death, elevated stomach pH, and an imbalance in the gastric microbiota caused by Hp infection lower Hp levels and allow non-Hp bacteria to colonize. Long-lasting inflammation causes DNA damage, gastric epithelial cell apoptosis, and autophagy, which in turn damages the gastric mucosa and causes GC and Hp-related gastropathy [34]. Hp is much less abundant in tumor tissues than in nearby non-tumor tissues, as it prefers to colonize healthy gastric mucosa [35, 36]. There are notable variations in the gastric microbiota between Hp-infected and non-infected patients, indicating that Hp might be involved in other microbial dysbiosis [37–40]. Other microbes become more prevalent as Hp abundance declines [41]. There were no discernible differences in the gut microbiota composition of Hp-positive and Hp-negative individuals in patients with late-stage gastric cancer [42, 43]. While Pseudomonas was significantly more common in tumor tissues, Hp and Lysobacter were significantly more common in normal tissues [44]. Hp plays a role in oncogenesis at the gastric level through three mechanisms [45]. First, Hp injects two cytotoxins, VacA and CagA into the host cell, which activates oncogenic signal transduction pathways [46–48]. A tiny RNA molecule; Hpnc 4160 in Hp has the capacity to suppress the expression of both outer membrane protein (OMP) and CagA causing autophagy inhibition and consequently malignant transformation [49, 50]. CagA-positive Hp mediates dysregulation of multiple signaling pathways including the Wnt/β-catenin signaling pathway, PI3K/Akt, NF-κB signaling pathway, Shh signaling pathway, JNK signaling pathway, JAK/STAT3 signaling pathway, and ERK/MAPK signaling pathway [51]. Second, it causes reactive oxygen species (ROS) to be produced, which in turn triggers inflammatory pathways. Finally, atrophic gastritis is characterized by the destruction of the parietal cells that produce acid so that there is a compensatory upregulation of gastrin that stimulates the cells to produce more acid, but also activates oncogenic signals. Following diminish of gastric acidity, the carcinogenic strength of a few bacterial lines might additionally increase. Additionally, the microbiota is likely populated with microorganisms that has the ability to form nitrites and carcinogenic N-nitroso compounds [52–55]. The USF1 gene has an important shielding function in Hp carcinogenesis and can be used potentially as a marker for susceptibility to GC [56, 46]. Watanabe et al. [57, 47] found that Hp infection was associated with reduced richness and evenness of gastric bacteria, and eradication of Hp only partially restored microbial diversity.

### Bacteria other than Hp and GC

Although other stomach microbes might have an impact on gastric carcinogenesis, their precise function is still unknown and needs more research [58]. The ‘Hp initiation–non-Hp acceleration’ cascade is increasingly recognized [59].

Gastric fluid samples from GC patients had larger amounts of Lactobacillus and Veillonella compared to lower levels of Verrucomicrobia and Deferribacteres [60]. There have been reports linking the phylum Verrucomicrobia, which includes Akkermansia, to the advancement of GC [61]. Fusobacterium was reported to be enriched in gastric adenocarcinoma [36, 62, 63].

Protumorigenic bacteria (e.g., E. coli and F. nucleatum) which are not predominant species in fecal microflora are enriched in the cancerous tissues, and may promote tumorigenesis by expression of genotoxins and virulence factors. E. coli, F. nucleatum, and B. fragilis by using experimental models are Potential tumorigenic pathobionts [64]. Mucosal colonization of Adherent-invasive E. coli (AIEC) through fimbriae-mediated adhesion was a crucial step for its colitogenic ability as a pathobiont [65]. Aside from causing genotoxicity, the induction of tumor-infiltrating macrophages and other unknown mechanisms may also play indispensable roles in E. coli-driven tumorigenesis. Enrichment of F. nucleatum was demonstrated in the stool and tissue samples of CRC patients [66]. The inconsistent data of F. nucleatum suggested that other unidentified mechanisms, such as interaction with other bacteria, may partly contribute to its protumoral characteristics. Entertoxigenic Bacteroides fragilis (ETBF) enterotoxin known as fragilysin acts as a metalloprotease that causes oxidative DNA damage, E-cadherin cleavage, epithelial barrier damage, and activation of STAT3/Th17 immune responses, and generation of protumoral monocytic myeloid suppressor cells [67]. Also, fragilysin stimulated the production of spermine oxidase in intestinal epithelial cell lines, suggesting a direct role of the enterotoxin on epithelial free radical production and DNA damage [68]. So, ETBF promote tumorigenesis through both direct and indirect mechanisms.

The gut microbiome of patients with esophageal cancer (EC), GC, and CRC differs from those of healthy people. The abundance alteration of R. faecis in patients with GI cancer might be a predictor of chemotherapy efficacy. Bifidobacterium, Ruminococcus and Roseburia; a member of the Clostridium coccodis cluster of the phylum Firmicutes, are considered a protective taxa in patients with EC, GC, and/or CRC and might be useful in identifying novel diagnostic biomarkers [69, 70]. The clinical chemotherapy response was not significantly associated with baseline microbiota. Moreover, no bacterial differences between responders and non-responders were observed in the patients with EC, GC, and CRC [70].

Results on microbial diversity were inconsistent. After analyzing nearly 200 gastric mucosal samples, Olabisi and his colleagues reported variations in the microbial composition without a discernible difference in microbial diversity [62]. Francisco et al., on the other hand, reported a decline in bacterial diversity from intestinal metaplasia (IM) to intestinal-type GC after non-atrophic gastritis (NAG) [58, 60, 61, 71]. This could be caused by a variety of factors, such as diet, ethnicity, sequencing methods, and the fact that the study populations included both Hp-positive and Hp-negative people. The analysis of different microbial groups in variable gastric regions can explain the heterogeneous results. There are differences between the proximal and distal GC in terms of microbial composition and metabolic products, despite the fact that there is no significant difference in species variety and abundance [72]. Clinically, EBV-associated GC (EBVaGC) is more common in men than women, tends to be found in the proximal region [73], has a better prognosis [74] and a comparatively low rate of lymph node metastasis [75].

The mechanism by which microbes and their metabolites affect gastric carcinogenesis is generally unclear. S. anginosus is consistently observed in mucosa biopsies of patients with GC and produces proinflammatory cytokines. The surface protein TMPC on S. anginosus mediates its attachment and colonization of gastric tissues. Activation of the oncogenic pathway can take place by the interaction of TMPC with the annexin A2 receptor on gastric epithelial cells. Compared with Hp which is mostly depleted in GC, S. anginosus is consistently involved in different stages of gastric carcinogenesis from precancerous lesion, mucosal atrophy, intestinal metaplasia, gastric dysplasia cascade, and finally to malignancy [76].

### Pathogenic fungus and virus in gastric carcinogenesis

Fungus sequencing showed that each tumor cell had a single fungus microbe. These microorganisms are less common in the esophagus and somewhat more common in the head and neck, colorectal, and stomach tissues [77]. The organization of fungal communities is drastically changed during gastric carcinogenesis, and the species richness, variety, and evenness of fungal components decline. Ascomycetes was the most enriched phylum in GC tissues, in contrast to the normally lower enrichment [78]. It is unknown if GC is caused by or follows from fungal dysbiosis [79]. Pu and his colleagues have recently emphasized the significance of age in determining the differences in the gut mycobiome. They further emphasize that the gut mycobiome of long-lived people has particular signatures that set them apart from other seniors. These signatures include an increase in core taxa and an overrepresentation of the Candida enterotype. Crucially, gut bacterial compositions are also strongly connected with these longevity-associated traits, which may be used as biomarkers to distinguish long-lived individuals from others [80].

**Fig. 1:**
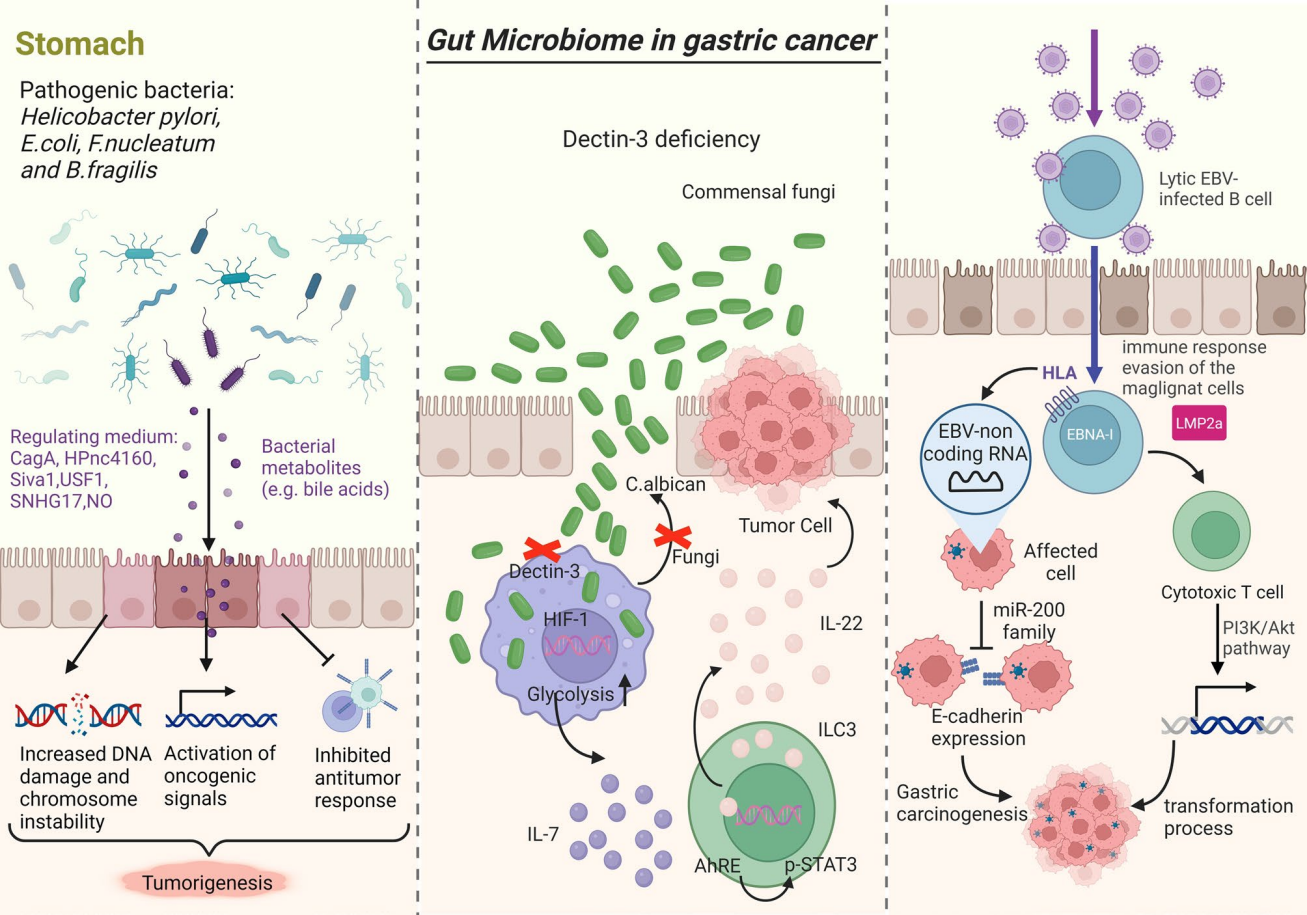
Proposed scheme of the role of gut microbiota disorders in the progression of GC. The left picture showed how bacterial pathogens promote tumorigenesis. Middle picture showed chromosomal instability, activate oncogenic signals, and suppress immune responses. Right picture showed EBV-non coding RNA is linked to the downregulation of the miR-200 family, resulting in decreased E-cadherin expression. Published by Mingjin Yang in Interaction between intestinal flora and gastric cancer in tumor microenvironment. Front. Oncol. 2024; 14:1402483. Doi: 10.3389/fonc.2024.1402483

Epstein-Barr virus (EBV) is one of the pathogenic viruses that has the ability to infect eukaryotic cells and produce cancer [81]. Other viruses such as human papillomavirus, human herpesvirus, and hepatitis virus showed no causal relationship and GC [82]. Figure 1.

### Mechanism of intestinal flora regulating tumor microenvironment

Through its impact on the tumor microenvironment (TME), the intestinal microbiome has a significant impact on the course and outcome of GC. The TME is primarily composed of tumor cells, stromal cells, immune cells, endothelial cells, and various secreted factors [83]. Because it interacts with tumor cells, promotes tumor growth and metastasis, and offers immune escape features, the TME’s immune cell population is extremely vital [84, 85]. Specific elements of the gut microbiota, such as distinct bacterial communities or distinct secretory factors, have a variety of functions in controlling immune cells in the TME, which affects the prognosis and results of cancer therapies [83].

#### The intestinal microbiota impacts most cancers development through modulating T-cellular activity

Hp infection can change the immune response during the chronic inflammatory phase by replacing CD8 + T cells with CD4 + T cells and changing the tissue-resident memory phenotype of CagA-specific CD8 + T cells [86]. Programmed death ligand 1 (PD-L1) can be expressed by gastric epithelial cells in response to Hp. These modifications might make it easier for Hp-infected cells to evade immune surveillance and develop into GC cells [87]. Certain bacteria can inhibit cancer cells by promoting T-cell activation; their absence or downregulation may subsequently aid in cancer development. Within the TME, microbiota can encourage the formation of tertiary lymphoid structures (TLSs), which are positive prognostic indicators for a range of solid tumors [88, 89].

#### The intestinal microbiota affects most cancers development through directing macrophage polarization

Tumor-associated macrophages (TAMs) have been classified into two subtypes: M1-like and M2-like [90]. M1-like macrophages stimulate type 1 helper T (Th1) cell immune responses, whereas they suppress type 2 helper T (Th2) responses. In contrast, M2-like macrophages are involved in Th2 immune responses and Th1 response inhibition, produce an extracellular matrix, and have anti-inflammatory characteristics [91]. TAMs in GC patients are closely linked to immune response changes and immune evasion by Hp [92]. Hp-macrophage interaction in the TME primarily consists of M2-like macrophage polarization induction, antigen presentation impairment, and macrophage secretion factor modulation, all of which promote GC invasion and progression [93].

#### The gut microbiota and other immune cells in the tumor microenvironment (TME)

Tumor growth in the TME is also facilitated by natural killer (NK) and dendritic cells (DCs). By impairing DC function, dysbiosis of the gut microbiota can increase tumor cell immune evasion. NK cells may have the ability to eradicate tumors after being influenced by the gut microbiota [83]. Intestinal flora can promote or inhibit tumor growth via bacterial products generation and interacting with TME through pattern-binding receptors. Bacterial genotoxins that cause DNA damage, genomic instability, and an increase in N-nitroso compounds, as well as bile acids, choline, neurotransmitters, and SCFA, are among the gene products and metabolites [94–96] from the gut flora that lead to chronic inflammation, impaired intestinal mucosal barriers, and altered immune responses. Lactic acid bacteria also produce lactic acid, which is used to create more harmful factors [97].The majority of the time, intestinal flora operates through pathogen-associated molecular patterns (PAMPs) and microbe-associated molecular patterns (MAMPs). This activates TLRs, whose signaling has been connected to the genesis of GC and are crucial for the innate immune response in the gastrointestinal tract. Myeloid differentiation factor-88 (MyD88), which is primarily involved in innate immune signaling that is triggered by Hp, is shared by most TLRs. GC invasion and migration may be impacted by TLR/MyD88 signaling, which regulates the expression of many cytokines and immune cells that infiltrate the TME [98].

### Tumor and Bacterial Extracellular Vesicles (BEV)

Bacterial extracellular vesicles (BEVs) are small molecule active substances formed from bacteria, characterized by longer circulation times and structural stability [99]. They play a crucial role in mediating interactions between bacteria and the host, impacting a range of physiological and pathological processes in both organisms [100] including the transport of virulence factors, biofilm formation, and antibiotic resistance [101].

BEVs have the potential to play a significant role in gastrointestinal cancer by infiltrating gastric mucosa and epithelial cells [102]. BEVs produced from host cells infected with Hp affect inflammatory signaling pathways, which in turn affect immune cell modification, cytokine release, cell proliferation, apoptosis, and endothelial dysfunction, cause cytoskeletal reorganization, damage cellular junctional structures, and significantly influence the course of subsequent immune-pathological reactions. These elements impact the course of GC and impede its pathogenesis [103]. Additionally, Hp liberates vesicles, referred to as outer membrane vesicles (Hp-OMVs), which contribute to atrophic and cell transformation in the gastric epithelium [104].

In order to promote antitumor immune responses in vivo for the treatment of cancer, BEVs can also act as strong immune stimulators. It has been demonstrated that OMVs released by bacteria cause anti-BFGF autoantibodies in tumor-bearing mice. These autoantibodies enhance tumor cell apoptosis, increase CTL responses, reverse tumor immunosuppressive microenvironments, inhibit tumor angiogenesis, and ultimately impede tumor growth [105]. By producing inflammatory mediators from gastric epithelial cells following their selective uptake by the cells, Hp EVs can cause inflammation and potentially cancer in the stomach [106]. Furthermore, studies by Li et al. demonstrate that BEV-derived HSP60 is essential for the emergence of Hp-related GC [107].

Via a biomimetic mineralization technique and utilizing a calcium phosphate coating on OMVs that dissolute in the acidic media upon arrival at the tumor location exposing the OMVs, this exposure successfully enhanced the tumor immune suppression microenvironment [108]. Guo et al. used BEVs as carriers to create a co-delivery system for chemical drugs [109].

With strong anti-tumor effects, E. Coli OMV may be a promising cancer immunotherapy agent [110]. Engineered OMV promotes the accumulation of effector T cells in tumors by a dual mechanism of checkpoint inhibition and immune activation [111]. Three intestinal bacterial strains were used to create novel nanovesicles (HNVs), which were linked to favorable immune checkpoint therapy outcomes. They also improve TME, encourage dendritic cell maturation and antigen presentation, and trigger innate immune activation [112]. OMV immunotherapy and HP DA-mediated phototherapy (PTT) were combined to increase the antitumor efficacy against melanoma. Combining PTT with the anti-tumor immune response greatly enhances treatment and results in the total eradication of melanoma [113]. Through extracellular vesicles, bacteria release bioactive metabolites that can change TME and selectively accumulate around tumor cells [114, 115]. TME-resident microbiota interactions are usually responsible for the presence of inflammatory carcinogenic metabolites in cancer cells [102]. Toxin-infected EV, which is released by certain intestinal bacteria, exacerbates inflammatory conditions and contributes to the development of CRC. They postulate that BEVs are crucial in the progression from inflammation to cancer [116]. BEVs are highly immunogenic and can be used as adjuvants, vaccines, and disease-treating vectors, particularly when delivering tumor antigens or small molecule drug-targeted treatments. BEVs are an emerging biomarker that can be found using liquid biopsy, providing new avenues for disease diagnosis [117]. In comparison to healthy controls, Kim et al. discovered that BEVs isolated from stool samples of CRC patients had a noticeably higher abundance of Firmicutes [118].

### Role of non-coding RNAs and gut microbiome in GI cancers

Non-coding RNAs (ncRNAs) have a pivotal role in gene expression, cancer progression, and cell-cell communication through the involvement of extracellular vesicles (EV). The gut microbiota controls the expression of microRNAs and abnormalities in their expression can result in pathogenic processes linked to the initiation and spread of cancer [119]. Crosstalk among microRNA-microbiota within the intestine performs a pivotal function in intestine homeostasis [120]. NcRNAs can act as tumor suppressors and oncogenes in cancers and they may be dealt with as promising diagnostic and healing markers. The microbiota can affect the occasion and development of most cancers by influencing the expression of ncRNA. MiRNAs play a vital role in the relationship in-between the host and the microbiota in cancer. Chang et al. in 2015 [121] showed that Hp-positive gastric cancer patients had considerably greater levels of miR99b-3p, miR-564, and miR-638 compared to Hp-negative patients, despite exhibiting significantly less miR-204-5p, miR-338-5p, miR-375, and miR-548c-3p. MiR-18a-3p and miR-4286 levels had been drastically large in gastric cancers related to Hp, following the analysis of Tsai et al. [79]. The gut microbiome and ncRNAs’ relation and pathway mechanisms are no longer known.

### Clinical implications

#### Gut microbiome and cancer prevention

It is now established that abnormal DNA methylation in non-cancerous tissues, namely mutation and epimutation loads, is linked to an increased risk of developing cancer [122, 123]. Several cross-sectional investigations found a correlation between the risk of gastric cancer and high methylation levels of several genes in the stomach mucosa [124]. Even when Hp is eradicated, the DNA methylation marker may be able to identify those who can avoid stomach cancer screening. Instead of being screened for GC every two years as is the current recommendation, the super-high-risk cohort identified by the DNA methylation marker will require screening annually [125].

Numerous studies have demonstrated that the microbiota and its metabolites can significantly influence anti-GC immunotherapy through cytokine release and increased T-cell infiltration [126]. Because antibiotics affect the gut microbiota, which plays a crucial role in enhancing the body’s immune response against tumors, their use reduces the effectiveness of cancer immunotherapy. Hp infection is a reliable predictor of worse outcomes during immunotherapy and a good sign of increased PD-L1 expression. Hp infection may be utilized as a marker to assess the effectiveness of immunotherapy in GC patients through unknown pathways since it has the ability to suppress both innate and adaptive immune responses [127]. By infiltrating CD8 + T-cells, the gut microbiota can affect how melanoma patients react to anti-PD-1/PD-L1 therapy. Additionally, they frequently have better progression-free survival [128]. Mice given probiotics along with antibiotics displayed improved 5-FU’s anti-tumor properties [129]. Oh et al. claim that using probiotics in addition to Hp eradication treatment attenuated the alteration of gut microbiota brought on by antibiotics, which may have decreased the risk factors associated with the emergence of GC [130]. In a mouse model of colitis-associated CRC, Song et al. [131] found that pretreatment with the probiotic mixture Bific altered the composition of the gut microbiota and reduced the expression of proinflammatory genes, improving colitis and reducing the formation of tumors. Turati et al. [132] discovered in a case-control study that eating a lot of galacto-oligosaccharide raffinose was associated with a lower incidence of GC. Using antibiotics to eradicate Hp may trigger the dysbiosis process. According to Watanabe et al. [133], dysbiosis may continue long after Hp is eliminated. Probiotic therapy significantly and persistently improved immune responses and reduced inflammation in patients who had partial gastrectomy [134]. By causing damage to the stomach mucosa, bile acids play a crucial pathogenic role in the development of Precancerous lesions of GC (PLGC). PLGC lesions of can be prevented and treated in the majority of cases by protecting the function of the gastric mucosa through the promotion or inhibition of specific mechanisms within the bile acids-gut microbiota interaction pathway [135].

#### Gut microbiome and treatment response

ICIs are a kind of cancer immunotherapy in which immune cells are reenergized to launch a powerful anticancer attack by antibodies that inhibit ICI molecules. Blocking antibodies against lymphocyte activation gene 3 (LAG3), cytotoxic T-lymphocyte associated protein 4 (CTLA-4), programmed cell death protein 1 (PD-1), and programmed cell death ligand 1 (PD L1) make up the currently approved ICIs [136]. The effectiveness of ICIs is influenced by the gut microbiota. The microbiome of cancer patients who respond to ICIs differs from that of individuals who do not [137]. Although ICI has completely changed the way that GC is treated, only 11–15% of patients respond overall. Therefore, it is essential to determine which patients may benefit from immunotherapy beforehand using non-invasive techniques. According to a 2024 study by Gao et al. [138], Dorea formicigenerans and Akkermansia muciniphila were important in correctly predicting the effectiveness of immunotherapy. They came to the conclusion that gut microbiome-based therapies might offer an option to boost immunotherapy’s efficacy. Gastrointestinal bacteria can directly or indirectly establish three distinct clinical outcomes: enhancing therapeutic side effects, avoiding anticancer effects, or promoting treatment efficacy [139]. The gut microbiota is important for the gut microbiota-immune axis and can affect the effectiveness of immunotherapy in several ways [140]. Treatment with 5-Fluorouracil (5-FU) was less effective when antibiotics were used [129]. Probiotics did not considerably improve the efficacy of the treatment as compared to 5-FU alone. Zheng et al. [134] found that a probiotic mixture comprising Bifidobacterium infantis, Lactobacillus acidophilus, Enterococcus fecalis, and Bacillus cereus decreased inflammation, improved immunity, and improved gut microbial balance in GC patients who had undergone partial gastric surgery. Han et al. [140] found that in patients with HER2-poor GC, the appearance of the gut microbiota influences the efficacy of several therapies (chemotherapy, immunotherapy, and combination therapy). Both the progression-free survival (PFS) and the results of anti-PD-1/PD-L1 immunotherapy are enhanced by the boosted Lactobacillus levels.

#### Potential microbial interventions and future directions

##### Prebiotics

These are substrates that host bacteria ferment and use specifically to provide health benefits [141]. The production of SCFAs can be increased by supplementing with Lycium barbarum polysaccharides. Additionally, the relative abundances of Bacteroidaceae, Lactobacillaceae, Prevotellaceae, and Verrucomicrobiaceae were favorably correlated with immunological characteristics, which improved the effectiveness of chemotherapy [142]. There is currently insufficient evidence to support the use of prebiotics in the clinical patient population to combat cancer.

##### Postbiotics

These are metabolites that can enhance health by promoting the microbiota’s metabolic activities [143].One classic example of substances which has been shown to have anticancer potential is SCFAs [144].Moreover, SCFAs have been associated with an aggressive PD1/PDL1 response in a variety of GI cancer types [145].The gut microbiota’s tryptophan metabolites show great potential apostbiotic supplements. The dietary tryptophan catabolite indole-3-aldehyde that Lactobacillus reuteri can release enhances the effectiveness of ICI therapy [146].

##### Antibiotics

The incidence of stomach cancer can be significantly reduced by removing Hp infection, a major risk factor for the carcinogenesis of GC [147]. Antibiotic medication can improve the efficacy of cancer treatment by lowering the therapeutic resistance brought on by microbiota [148].Antitumoral immunity is produced by bacterial elimination, which creates microbial neoantigens that share host-specific epitopes with the host [149].

##### Phage therapy

Phage-based drug development signifies a revolutionary advancement in contemporary medicine, going well beyond conventional phage therapy for bacterial infections. This method leverages the adaptability of bacteriophages for a diverse array of uses, including cancer treatment, vaccine creation, and drug-delivery systems (DDS). By modifying phages to specifically target disease markers, transport therapeutic substances, or provoke immune responses, researchers are finding innovative approaches to tackle intricate medical issues, such as improving therapeutic effectiveness, addressing a variety of pathogens, and surmounting conventional drug-delivery obstacles. Phage therapy utilizes viruses that specifically attack bacteria to treat infections. This technique has demonstrated success in numerous clinical instances, especially for patients suffering from severe infections caused by bacteria resistant to multiple drugs. For instance, there have been instances of individuals suffering from systemic infections due to multidrug-resistant Acinetobacter who experienced recovery after receiving phage treatment, as well as patients afflicted with panresistant Pseudomonas aeruginosa who were successfully treated with phage therapy. A notable characteristic of phage therapy is its remarkable specificity. Phages selectively infect particular bacteria and eliminate them. However, this specificity may also pose a challenge, as it requires meticulous selection of the right phage for the targeted bacteria. Moreover, bacteria have the potential to develop resistance to phages, which could diminish the effectiveness of treatment over time. Efforts are being made to identify, combine, and enhance phages to tackle these issues. The use of phage therapy in clinical settings is crucial for providing life-saving options for patients facing severe bacterial infections, and combining it with antibiotics may improve therapeutic outcomes [150, 151]. Phage therapy offers a hopeful method for treating cancer, providing targeted and diverse strategies to combat different forms of the disease. However, despite their potential, phage-based cancer treatments encounter various obstacles that need to be overcome to fully harness their advantages.

##### Drug delivery system

Because of their unique characteristics, such as hypoxia tropism, certain microorganisms can be engineered to specifically target the hypoxic tumor tissues [152]. Myeloid-derived suppressor cells (MDSCs) are susceptible to infection by Listeria species, which then deliver the bacteria to the tumor sites and allow them to migrate from MDSCs into tumor cells [153]. Moreover, Listeria species can be engineered to deliver anticancer drugs by targeting tumors [154].

##### Genetically engineered microorganisms

Many research studies have demonstrated that bacterial-based therapeutic approaches are effective in treating cancer, notably reducing tumor growth and stimulating the immune response, all while ensuring a high level of safety and enhancing patient survival rates [155]. The existence of bacteria within tumors has been acknowledged for a long time, but their exact source remains uncertain. Three possible origins for the bacteria associated with tumors have been suggested: bacteria from mucosal locations that can breach the mucosal barrier and reach the tumor, bacteria from nearby healthy tissues, and bacteria that travel to the tumor site through the bloodstream. Bacteria have a multifaceted role in the process of tumor progression, potentially functioning as both enhancers and inhibitors of tumor growth. The ways in which tumor-associated microorganisms affect tumor progression are intricate and often contradictory. Substantial alterations can be made to modify surface composition and structure, decrease toxicity, or introduce therapeutic agents through genetic engineering. These adjustments allow bacteria to be modified into low-toxicity, high-efficiency micro/nanobots targeting tumors. With the help of chemical, physical, and genetic engineering techniques, bacteria have surpassed their inherent therapeutic limitations, evolving into efficient delivery vehicles for a range of therapeutic drugs, functioning like precise “couriers” that convey medications directly to the tumor location [156, 157]. Bacteria can be genetically tailored to transport a diverse array of personalized therapeutic agents, such as prodrug-converting enzymes, cytotoxic agents, immune modulators, cytokines, small interfering RNAs (siRNAs), and nanobodies [158–163]. Applications of engineered bacteria in cancer therapy include Living bacteria cancer-targeted therapy via metabolic modulation, engineered bacterial cancer-targeted therapy via synergistic approaches, engineered bacteria cancer-targeted therapy with photodynamic therapy (PDT), engineered bacteria cancer-targeted therapy with photothermal therapy (PTT), engineered bacteria cancer-targeted therapy with chemotherapy, engineered bacteria cancer-targeted therapy with radiotherapy [164].

Gene therapy, which holds great promise for the treatment of cancer problems, has been implemented using several viral and non-viral gene delivery strategies. To transfer anticancer genes to areas with tumor hypoxia, for example, the gut probiotic E. coli Nissle 1917 has been adapted to act as a targeted transport vector [165]. By genetically altering the arginine inhibitory gene in E. coli Nissle 1917 to alter the quantity of L-arginine in tumors, PD-L1 immunotherapy can be made more effective [166].

##### Advantages and challenges of microbial interventions

By enhancing risk-adapted therapy options and aiding in the stratification of cancer patients with differing degrees of severity, the development of an accurate microbiome-based evaluation regimen may lower cancer mortality [167–169]. Regional variances and microbial alterations show the largest connections. These regional variations restrict the extrapolations of a limited number of diagnostic models between districts, according to He et al. in 2018 [170]. This suggests that it is essential for clinical investigators to appropriately represent the information of disease models that generate reference data.

##### Probiotics safety

According to certain case studies, taking live probiotics may cause a variety of negative side effects, such as sepsis, pneumonia, abscess, meningitis, and endocarditis [171]. As a result, each probiotic strain’s hazards and risk/benefit ratios must be thoroughly assessed in clinical practice. Since only a few genera of probiotics have been shown to have beneficial effects, it is critical to screen and identify the strains that genuinely aid in therapy [172, 173].

##### Prebiotics/Postbiotics concern

Prebiotics and postbiotics are safer and less likely to cause negative effects because they do not contain living bacteria. Consuming adequate dietary fiber is considerably more beneficial for cancer patients during ICI treatment than probiotic use [174]. Gut bacteria react differently to dietary prebiotics because different fermentative routes are imposed on microbial collection [175]. According to Singh et al. in 2018, SCFAs may increase the risk of hepatocellular carcinoma in some circumstances despite their anticancer impact [176].

##### Fecal microbiota transplantation-related adverse events

In total, 19% of significant incidents connected to FMT took place. The majority of these were gastrointestinal problems, such as diarrhea (10%) and abdominal pain, discomfort, and cramping (7%). Only 1.4% of people experience serious side effects from FMT, including infections and death. This particular data comes from a population with many different conditions, but none of them are specifically for cancer patients. Patients with mucosal barrier damage are the only ones who experience serious side effects from FMT. To lower the risk of side effects, colonoscopy tests must be performed both before and after FMT treatment [177]. Washed microbiota transplantation (WMT) improves quality control, safety, and accuracy. The washing process removes the harmful substance [178]. Recipient parameters, not donor factors, determine strain dynamics particular to a species after FMT [179]. Strict protocols must also be adhered to while screening FMT recipients in order to protect donors and patient safety.

##### Antibiotic-related concern

As of right now, there is ongoing debate on the use of antibiotics in cancer treatment. Certain antibiotic therapies can inhibit the growth of cancer brought on by microbial infections or dysbiosis [180] and reverse therapy resistance brought on by microbiota [148, 181, 182]. Antibiotic treatments, however, may disturb the gut ecology due to their detrimental effects on the native microbiota, which can result in a loss of diversity and notable alterations in the microbial community’s composition [140]. They may also reduce the efficacy of chemotherapy [183, 184], radiation [185], and immunotherapy [186]. Antibiotics decrease their efficiency and raise the risk of illness by making microorganisms resistant to them [187].

## Conclusion

Through the use of impacting TME, the intestinal microbiota plays a crucial role in influencing the formation and analysis of GC. Through the release of extracellular vesicles, the gut microbiota exchanges information between cells. Liquid biopsies, which are microRNAs, have recently demonstrated their enormous potential as novel biomarkers for the majority of cancer diagnosis. A crucial and changeable component of the majority of cancer treatments is the gut flora. NcRNAs are intriguing avenues for future therapeutic and diagnostic research. Further research is required to completely understand the involvement of ncRNAs in GI cancer. By modifying drug metabolism and absorption, which have a substantial impact on healing efficacy and serious impact profiles, the dynamic changes in gut microbiota also have a considerable impact on the results of anti-cancer treatment. Bacterial extracellular vesicles change the tumor microenvironment, which may impact medication resistance, metastasis, immunosuppression, and the course of cancer. Improved treatment approaches may arise from a better understanding of the role of the microbiome in gastric malignancies.

## Data Availability

No datasets were generated or analysed during the current study.

## Abbreviations

GC: Gastric cancer
IM: Intestinal metaplasia
AG: Atrophic gastritis
NAG: Non-atrophic gastritis
Hp: Helicobacter pylori
SCFAs: Short chain fatty acids
OMP: Outer membrane protein
ROS: Reactive oxygen species
EBV-aGC: EBV-associated GC
TME: Tumor microenvironment
PD-L1: Programmed death ligand 1
PD1: Programmed death 1
TLSs: Tertiary lymphoid structures
TAMs: Tumor-associated macrophages
NK: Natural killer
DCs: Dendritic cells
PAMPs: Pathogen-associated molecular patterns
MAMPs: Microbe-associated molecular patterns
MyD88: Myeloid differentiation factor-88
NcRNAs: Non-coding RNAs
MiRNA: MicroRNAs
BEVs: Bacterial extracellular vesicles
FMT: Fecal microbiota transplantation
WMT: Washed microbiota transplantation
